# A systematic review of patient and health system characteristics associated with late referral in chronic kidney disease

**DOI:** 10.1186/1471-2369-9-3

**Published:** 2008-02-25

**Authors:** Sankar D Navaneethan, Sarah Aloudat, Sonal Singh

**Affiliations:** 1 Division of Nephrology, University of Rochester, Rochester, NY, USA; 2 Department of Medicine, University of Texas at Houston, TX, USA; 3 Department of Medicine, Wake Forest University School of Medicine, Winston-Salem, NC, USA; 4 MPH Program, Bloomberg School of Public Health, Johns Hopkins University, Baltimore, MD, USA

## Abstract

**Background:**

To identify patient and health system characteristics associated with late referral of patients with chronic kidney disease to nephrologists.

**Methods:**

MEDLINE, CENTRAL, and CINAHL were searched using the appropriate MESH terms in March 2007. Two reviewers individually and in duplicate reviewed the abstracts of 256 articles and selected 18 observational studies for inclusion. The reasons for late referral were categorized into patient or health system characteristics. Data extraction and content appraisal were done using a prespecified protocol.

**Results:**

Older age, the existence of multiple comorbidities, race other than Caucasian, lack of insurance, lower socioeconomic status and educational levels were patient characteristics associated with late referral of patients with chronic kidney disease. Lack of referring physician knowledge about the appropriate timing of referral, absence of communication between referring physicians and nephrologists, and dialysis care delivered at tertiary medical centers were health system characteristics associated with late referral of patients with chronic kidney disease. Most studies identified multiple factors associated with late referral, although the relative importance and the combined effect of these factors were not systematically evaluated.

**Conclusion:**

A combination of patient and health system characteristics is associated with late referral of patients with chronic kidney disease. Overall, being older, belonging to a minority group, being less educated, being uninsured, suffering from multiple comorbidities, and the lack of communication between primary care physicians and nephrologists contribute to late referral of patients with chronic kidney disease. Both primary care physicians and nephrologists need to engage in multisectoral collaborative efforts that ensure patient education and enhance physician awareness to improve the care of patients with chronic kidney disease.

## Background

Chronic kidney disease is an emerging public health problem. A recent study reported that nearly 26 million Americans suffer from Chronic Kidney Disease (CKD) [1]. Future projections for the US population estimate more than 700,000 prevalent cases of End Stage Renal Disease (ESRD) by the year 2015 [2]. Observational studies and their meta-analysis have shown that late referral of patients with CKD to nephrologists is associated with poor clinical outcomes [3-7]. Longer pre-dialysis care by nephrologists may result in reduction in rates of hospitalization and mortality [3-7].

There is no universally accepted definition of 'Late referral' of patients with CKD. Several inconsistent criteria including the number of months prior to the initiation of dialysis(1 month, 3 months or 6 months), or the stage of CKD have been used to define late referral of patients with CKD. The National Kidney Foundation-Kidney Disease Outcomes Quality Initiatives (K-DOQI) guidelines recommend that patients with CKD be referred to nephrologists when the glomerular filtration rates (GFR) fall below 30 ml/min (Stage 4 CKD), and earlier if possible (evidence category-opinion) [8]. Similar guidelines have been issued by other agencies [9-11]. This inconsistency in the definition of late referral is attributable to changing practice patterns among physician's, changes in the definition of CKD, and increasing awareness among physicians and patients. The optimal timing of referral varies depending on physicians' characteristics and preferences, practice setting, the comfort level of the treating physicians and the availability of nephrologists.

The development of interventions to address late referral of patients with CKD is hampered by the lack of a comprehensive understanding of the factors responsible for late referral. Despite the existence of these guidelines, nearly 15–80% of patients who start dialysis are referred late [12-14]. Retrospective studies and narrative reviews have identified several individual factors, such as ethnicity and insurance status that contribute to late referral [15-20]. Late referral now documented for over 15 years is increasing as reported in recent studies.

Our objectives were to systematically review the evidence on patient and health system characteristics associated with late referral of patients with CKD. In order to be comprehensive in our approach, we aimed to ascertain the factors associated with late referral as defined in earlier studies (<1 month prior to initiation of dialysis to <6 months of initiation of dialysis), as well as the definition of late referral outlined in the current National Kidney Foundation guideline recommendations (patients with GFR below 30 ml/min or in Stage 4 CKD).

## Methods

### Search Strategy

In March 2007, we searched MEDLINE, CINAHL, and CENTRAL for epidemiological studies using the following search terms: "Referral and Consultation" [MeSH] AND "Kidney Failure, Chronic" [MeSH], *delayed referral*, *late referral*, *chronic kidney disease*, *nephrologists*, *physician*, *reasons and causes*. We used the "Related Articles" link in PubMed and reviewed the references of identified studies for additional studies. Our search was limited to studies published in the English language. We included both prospective and retrospective observational studies, along with physician surveys. Studies in which adult patients were referred within 6 months before initiation of dialysis, or referred to nephrologists in stage 5 CKD were eligible for inclusion. We excluded studies enrolling patients less than 18 years of age.

**Figure 1:**
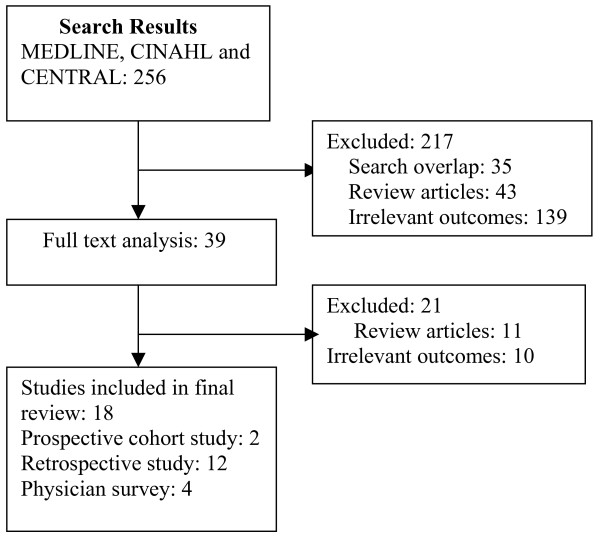
Flow chart showing the search, the major reason for exclusions of studies and the final number of studies included in this review.

The initial search resulted in 256 potential articles. Two reviewers (SDN, SA) independently and in duplicate reviewed the abstracts of 256 articles, and determined their eligibility for inclusion based on prespecified inclusion criteria as identified above. Two hundred and seventeen citations were excluded as they were review articles, non-English citations or analyzed the outcomes of late referrals. Thirty-nine full text articles were reviewed, and 21 were excluded as they were review articles or analyzed the outcomes of the late referrals. Eighteen studies were included in the final review [15-18,21-34] (Figure 1). Data extraction and content appraisal were done using a standard data extraction form. Disagreements were resolved by discussion with SS.

For the sake of clarity of understanding, we separated the reasons for late referral into two categories: 1) Patient related (including disease related factors i.e. patients suffering from an acute onset of kidney disease or deterioration of their CKD or asymptomatic kidney disease) and 2) Health system related. These two categories require different preventive approaches and specific interventions. A meta-analysis could not be performed as odds ratio or risk ratios were not consistently reported in the studies.

### Characteristics of Included Studies

Two groups of studies were identified. The first group of studies included patients on dialysis while the second group of studies addressed late referral through physician surveys.

Most studies of patients on dialysis were retrospective, except for two larger prospective studies conducted in US and Europe [15,18]. The reasons for late referral were analyzed using dialysis records, and physician visit records [15-18,21-30]. The duration of care provided by nephrologists varied from 1–12 months in these studies. The included studies varied in population selection, exclusion vs. inclusion of patients who had "inevitable" late referral (i.e. patients with an acute cause of ESRD who did not have the opportunity for timely evaluation by a specialist), and whether they were population-based vs. single center studies. Some limited their studies to the elderly population. The number of participants in these studies ranged from 135–3334. The ascertainment windows for the definition of patient and/or health care system-related characteristics, and health-care environment (e.g., European-style universal health-care coverage vs. highly uninsured inner-city patients in Manhattan) varied widely across studies. Winkelmayer identified factors predicting late referral from a retrospective review of Medicare and Medicaid database in the state of New Jersey [26]. Wauters et al analyzed the predictors of late referrals in 279 patients with universal health coverage in Europe [28]. Other characteristics are outlined in the Table 1.

**Table 1:** Characteristics of studies that identified patient and health system related factors in the reasons for late referral of patients with chronic kidney disease.

**Study**	**Year**	**Type of study**	**Setting/Country/Number of participants**	**Definition of Late referral (months prior to start of dialysis or stage of CKD)**	**Patient related factor**	**Health care related factor**
Arora [23]	1999	Retrospective	Tertiary referral center/USA/135 dialysis patients	<4 months		●
Cass [22]	2003	Retrospective	Australian and New Zealand dialysis and transplantation registry/3334 patients	<3 months		●
Ifudu [28]	1999	Retrospective	Tertiary referral center/USA/220 dialysis patients	Creatinine > 4 mg/dl	●	
Jungers [16]	1993	Retrospective	Single dialysis center/France/354 patients	<1 month	●	
Khan [21]	1994	Retrospective	Renal infirmary/UK/304 CKD patients	NA	●	
Kinchen [15]	2002	Prospective cohort	Dialysis centers/USA/828 dialysis patients	<4 months	●	●
Navaneethan (30)	2007	Retrospective	2 Dialysis units/USA/205 patients	<6 months	●	●
Obialo [29]	2005	Retrospective	Tertiary referral center/USA/460 patients	<3 months-Late referral <1 month-ultra late referral	●	
Roderick [25]	2002	Retrospective	Tertiary referral center/UK/250 dialysis patients	< 4 months		●
Schmidt [17]	1998	Retrospective	3 Dialysis units/USA/238 patients	<1 month		●
Steel [27]	2002	Retrospective	Single dialysis center/UK/494 dialysis patients	< 3 months	●	
Winkelmayer [26]	2001	Retrospective	New Jersey ESRD data/USA/3014 dialysis patients	< 3 months	●	●
Wauters [18]	2004	Prospective cohort	Multiple dialysis centers/Europe/279 dialysis patients	<6 months	●	●

The studies that addressed late referral through physician surveys were surveys predominantly conducted in North America and analyzed physicians' response to clinical scenarios [31-34]. Five studies identified only patient related factors [22,23,29,31,34], nine studies identified only health system related factors [15,17-20,24-26,32,33], and four studies identified both patients and health system related factors [16,21,27,28] associated with late referral. Other characteristics are outlined in the Table 2.

**Table 2:** Characteristics of physician studies that identified factors associated with late referral of patients with chronic kidney disease.

**Study**	**Year**	**Type of study**	**Country/No. of physicians**	**Types of physicians**	**Referral predictors identified**
Boulware [34]	2006	Physician Survey	USA/304	Family practioners/Internists/Nephrologists	Insurance status, non-compliance
Campbell [33]	1989	Physician interview	UK/86	General physicians	Lack of specific referral criteria, Patient refusal, Physician rationing
Lameire [24]	1999	Survey of dialysis units	Europe/NA	General physicians/Internists/Specialists	Physician rationing based on age, presence of comorbidities
Mendelssohn [32]	1995	Physician survey	Canada/728	Family practioners/Internists	Increasing age, presence of comorbidities, shorter life expectancy
Parry [31]	1994	Physician survey	UK/203	Geriatricians/General physicians/Nephrologists	Increasing age, presence of comorbidities, availability of dialysis centers

## Results

### Patient Characteristics

Eight studies identified age, race, gender, comorbidity, etiology of renal disease, and patient non-compliance as being characteristics associated with late referral of patients with CKD.

### Age and Gender

The increasing age of patients was associated with late referral in several studies conducted in North America. Winkelmayer et al showed that age over 75 years was associated with late referral (Odds Ratio (OR), 1.73, 95%CI 1.44–2.08). This association was more significant in patients over 85 years (OR, 2.66, 95%CI, 1.87–3.79) when compared to patients between the ages of 65–74 years (referent group) [26]. Ifudu et al concluded that even patients aged > 55 years were referred late (OR, 4.7, 95% CI 1.37–16.0) when compared to patients aged < 55 years [28]. Our previous study showed that age > 75 years was found to be significantly associated with late referral among patients in a community in New-York in comparison to patients aged < 75 years (*P *= 0.03) [30]. A physician survey conducted in Canada concluded that primary care physicians are less likely to refer older patients to nephrologists than younger patients [32]. In contrast, studies by Jungers et al, Wauters et al, Steel et al, and Arora et al did not find any age differences in patients with CKD who were referred early compared to those who were referred late [16,18,23,27].

Winkelmayer et al showed that being male nonsignificantly increased the odds of late referral (OR, 1.16, 95% CI-0.99–1.37) [26]. Several other studies reported no gender differences in the referral of patients with CKD[16,18,30].

### Race

Winkelmayer et al identified a significant association between race other than black or white and late referral (OR, 1.68, 95% CI 1.21–2.32)[26]. Kinchen et al, and Ifudu et al showed that black and Hispanic patients were referred late [15,28]. In contrast, Steel et al from UK concluded that whites might be referred later to nephrologists than blacks, although the results were non-significant (p = 0.08) [27]. In contrast Jungers et al, Arora et al and our own study did not identify any association between race and late referral of patients with CKD[16,23,30].

### Comorbidity

The presence of comorbid illness was associated with late referral in most studies. Kinchen et al found that patients with higher index of coexistent disease score (combination of index of physical impairment and index of disease severity) were nearly twice more likely to be referred late than their counterparts with lower scores (OR, 1.8, 95%CI, 1.16–2.84) [15]. Similar results were seen in the largest European study [18,21]. Wauters et al concluded that presence of an active cancer would delay the referral of patients with CKD to nephrologists [18]. Khan et al allocated CKD patients to low, intermediate, and high-risk groups based on their age and the presence of other comorbidities (heart disease, diabetes and pulmonary disease)[21]. The presence of these coexisting illness resulted in late referral. A physician survey by Mendelssohn et al identified that the presence of comorbidity would result in late or non-referral by physicians [32]. In an earlier study, we determined that patients referred late had a higher Charlson Comorbidity Index (calculated with 17 comorbidities) than patients referred earlier (OR, 1.17, 95% CI, 1.04–1.32, *P *= . 009) [30]. In contrast, Winkelmayer determined that the presence of hypertension (OR, 0.47 95% CI, 0.40–0.56), malignancy (OR, 0.73, 95% CI, 0.59–0.91), coronary artery disease (OR, 0.69, 95%CI, 0.58, 0.82) and diabetes (OR, 0.82, 95% CI 0.69–0.97) resulted in earlier referral to nephrologists in comparison to patients with no comorbidities [26]. Arora et al did not find an association between the presence of comorbidity and late referral, most likely as a result of local differences in practice patterns [23].

### Etiology of Renal disease

Only a few studies specifically explored the relationship between the etiology of renal disease and late referral. Patients with non-diabetic kidney disease were 1.4 times (95% CI 1.15–5.26) more likely to be referred later to nephrologists than patients with diabetic kidney disease in our previous study [30]. In the study by Jungers et al, patients with congenital kidney disease were referred earlier compared to patients with hypertensive renal disease [16]. Patients with rapidly progressing kidney disease were referred earlier in comparison to patients who had gradual worsening of renal function (OR, 7.1, 95%CI, 2.9–16.7) [16].

### Patient Non-compliance

Jungers et al showed that 42% of late referrals could be attributed to patient non-compliance [16]. However, the role of patient non-compliance could not be adequately assessed as most studies identified late referral of patients from dialysis records.

## Health system related

### Insurance status

Two large retrospective studies in USA showed an association between insurance status and late referral (15, 26). Kinchen et al concluded that uninsured dialysis patients in 81 larger dialysis centers were three times more likely to be referred late to nephrologists than patients with insurance (OR, 3.2, 95% CI, 1.45–7.07)[15]. Winkelmayer et al concluded that patients with Medicaid insurance were at a nonsignificantly higher risk of being referred late to nephrologists compared to patients with other insurance types in New Jersey (OR, 1.17, 95% CI, 0.98–1.39), [26]. In contrast, Arora et al concluded that patients covered by health care maintenance organizations (HMOs) were referred later than patients covered by Medicaid and the results were statistically significant (OR, 4.5, 95% CI, 1.3–14.6) [23]. However, these findings from a single center in a state that had a substantial HMO presence are not generalizable. Obialo at al showed that being homeless or unemployed was significantly associated with late referral and ultra-late referral (<1 month prior to dialysis initiation) (OR, 6.0, p-value = 0.004) [29]. Economical reasons were cited in one-fourth of these patients in their study.

### Type and location of referral and dialysis center

Wauters et al identified that late referral was more frequent in larger centers than in private or regional centers (OR, 7.3, 95% CI, 1.8–30) [18]. Schmidt identified no differences in referral pattern among patients living more than an hour away from dialysis units [17].

### Physician factors

Physician specialty, their knowledge of guidelines about timing of referral, and their perceptions towards outcomes of patients with CKD were analyzed in a few studies. Winkelmayer et al found that general internists, rather than family physicians or specialists were more likely to refer patients with CKD later to nephrologists [26]. Wauters et al concluded that specialists or primary care physicians were more likely to refer patients with CKD later as compared to family physicians [18]. Similarly, Lamiere et al found that specialists, rather than general practitioners are more likely to refer patient with CKD later in a large European study [24]. Campbell et al found that more than 90% of referring primary care physicians felt that they had inadequate training regarding timing or indications for referral of patients with CKD [33]. Recently, Boulware et al showed that primary care physicians identified patients with CKD later, performed lesser diagnostic work-up, and were statistically significantly less likely than nephrologists to recommend referral to a nephrologist (p < 0.01) [34]. Mendelssohn et al concluded that 'rationing by physicians about the need for dialysis' was a major factor in late referral [32]. Physicians evaluated the distance of dialysis centers and overcrowding of the nearest dialysis centers before referring a patient with CKD.

## Discussion

### Key Findings

Our systematic review demonstrates that a wide range of both patient and health system related barriers are associated with late referral of patients with CKD to nephrologists (Table 3). Overall, being older, belonging to a minority group, being less educated, being uninsured, and suffering from multiple comorbidities, and the lack of communication between primary care physicians and nephrologists contribute to late referral of patients with CKD.

**Table 3:** Patient and health system factors associated with late referral of patients with chronic kidney disease

**Patient related factors**	**Health system related factors**
Age	Insurance status
Race	Type of referring physician
Gender	Type of referring center
Comorbid illness	Physician rationing
Etiology of renal disease	Distance to dialysis center
Patient noncompliance	
Socioeconomic status	

Old age was consistently associated with late referral in several studies. One study showed that even age > 55 years is associated with late referral and several others showed age > 75 years as a major predictor of late referral [28]. Thus, it is prudent to assume that the risk for late referral increases, as one gets older. Despite varying criteria used to define minority racial groups across studies, belonging to "other" race was highly predictive of late referral in most studies. Black race was not associated with late referral in Winkemayer's study [26], due to their ability to adjust for comorbidities and multiple demographic factors, including socioeconomic status. Geographic differences in practice patterns may also explain some of these racial differences in the propensity to refer blacks and whites to nephrologists for renal replacement therapy.

Most studies that evaluated the association of insurance status with late referral failed to adjust for the socioeconomic status of patients with CKD. Both socioeconomic status and insurance status are inextricably linked. Patients in low socioeconomic status are at high risk for late referral as shown by Obiala et al [29]. Patients with CKD from densely populated areas with a predominantly indigenous population in Australia experienced more late referrals to nephrologists than other populations [22]. Jungers et al did not identify socioeconomic status as a risk factor in their study in France, because all geriatric patients are eligible for full health care coverage in France [16]. Even in countries, which provide for some degree of universal healthcare coverage, significant socioeconomic disparities persist across a range of health indicators and in access to healthcare as shown above.

The coexistence of multiple comorbid illnesses increased the risk of late referral of patients with CKD to nephrologists in several larger studies conducted in the United Kingdom, [21] United States, [15] and Europe [18]. However, in some smaller studies, the presence of comorbidity may have contributed to early referral. Patients with CKD and other coexisting illness may have their renal function monitored more frequently as part of routine chemistry panels. Early referral may reflect enhanced physician awareness of the relationship between these diseases and CKD progression, or increased physician attentiveness to management of patients with CKD, because of an increased frequency of patient-physician interaction. These seemingly contradictory results could be attributed to geographic variations, type of patients included, provider misconceptions about the outcome of patients with multiple comorbidities on dialysis.

### Limitations

Our review is subject to limitations inherent in a systematic review of a small sample of observational studies. Causality should not be inferred from these associations. The lack of a consensus definition for 'Late referral' may account for the inconsistent results across these methodologically diverse groups of studies. The risk ratios across studies are not comparable preventing us from making any inferences on the relative importance of these factors in contributing to late referral. It is difficult to ascertain the independent effect of closely linked factors, e.g. race, insurance and socio-economic status, which are more likely to exert their effects in combination. We could not determine the degree of publication bias but negative studies that fail to report an association of factors with late referral are less likely to be published. The majority of studies included in our review identified late referral of patients on dialysis, preventing us for determining the factors associated with non-referral of patients or those patients who failed to comply with their physicians recommendations.

Geographical similarities and differences in practice and organization of the health system need to be considered in interpreting our results. The survey conducted by Mendelssohn et al, among family physicians and community internists in Ontario, Canada showed that some patients with ESRD were not referred to a nephrologist [31]. This non-referral was influenced by age and coexisting disease [32]. Similar rationing by physicians was not demonstrated in a survey conducted in England [31]. Specialists (other than nephrologists) were likely to recommend referral for patients with CKD later than primary care physicians. This may not be relevant to the US where most patients with CKD are first seen by primary care physicians rather than specialists. Unfortunately, even primary care physicians referred patients with CKD later to nephrologists [34]. Primary care physician's referral patterns are similar to other specialists; this message is in contrast with most of the published observations in other specialties (primary care physicians referral patterns are similar to nephrologists and much better than other specialists, in particular internists and diabetologists).

There are no studies that specifically assessed whether patients with proteinuria with preserved GFR would benefit from early referral. However, referral of all patients with diabetes and proteinuria (with preserved GFR) may result in early referrals and longer waiting time for patients with advanced stage CKD. These questions need to be explored in future studies.

#### Implications

Our study has important implications for both clinicians and policy makers in the United States and abroad. Lack of provider knowledge about the appropriate timing of referral may account for over 25% of late referrals. The success of interventions to sensitize providers through automatic reporting of glomerular filtration rates when serum metabolic profiles are requested needs further investigation. Although automatic reporting of GFR has increased the identification of patients with CKD, it is unclear whether it increases the appropriate referral of patients of chronic kidney disease [35,36]. Early referrals may improve the quality of care of some patients, but may result in longer waiting time for other patients with advanced stage CKD given the national shortage of nephrologists in the US [35,36].

The National Kidney Foundations initiative to educate patients about chronic kidney disease through awareness of their 'kidney number' is commendable and may enhance patient and provider awareness [37]. Media related awareness programs about kidney disease need to be enhanced [38]. In the future, simple referral guidelines need to be prepared in collaboration with primary care physicians [39]. Co-management approaches for chronic kidney disease need to be evaluated [39].

Several factors associated with late referral of patients with chronic kidney disease – such as low socioeconomic status, insurance status, and educational status will require policy interventions. Policy interventions will need to address the both patient- and healthcare related barriers, especially as they affect access to health care for socioeconomically disadvantaged and ethnic minorities.

Future studies should analyze the impact of the guidelines on referral pattern, begin from the primary care office rather than at the dialysis end, and address the interplay and the relative importance of these factors. Prospective studies assessing the efficacy of a multi-sectoral, multimodality approach comprising of targeted educational interventions for physicians are needed. These approaches need to be especially geared towards physicians providing care for a population which is ethnically diverse, older and has multiple coexistent comorbidities. It remains to be seen whether efforts at increasing patient and provider awareness will translate into earlier referral, and ultimately better care of patients with chronic kidney disease.

## Conclusion

Overall, being older, belonging to a minority group, being less educated, being uninsured, and suffering from multiple comorbidities, and the lack of communication between primary care physicians and nephrologists contribute to late referral of patients with CKD. Policy maker need to address the health system barriers identified in our review. Both primary care physicians and nephrologists need to engage in multisectoral collaborative efforts that ensure patient education and enhance provider awareness to improve the care of patients with chronic kidney disease.

## Competing interests

The author(s) declare that they have no competing interests.

## Authors' contributions

SDN designed the study, performed the search, data extraction and wrote the manuscript. SA helped with the selection of studies, data extraction and writing the manuscript. SS helped with the design of the study, selection of studies and writing the manuscript. All the authors approved the final version of the manuscript.

## Pre-publication history

The pre-publication history for this paper can be accessed here:


